# A general method to fabricate MoO_3_/C composites and porous C for asymmetric solid-state supercapacitors†

**DOI:** 10.1039/c8ra10232e

**Published:** 2019-04-30

**Authors:** Yu Jiang, Xuemin Yan, Yapeng Cheng, Yan Zhang, Wei Xiao, Lu Gan, Haolin Tang

**Affiliations:** College of Chemistry and Environmental Engineering, Yangtze University Jingzhou 434023 Hubei China XueminYan@126.com; State Key Laboratory of Advanced Technology for Materials Synthesis and Processing, Wuhan University of Technology Wuhan 430070 PR China thln@whut.edu.cn; School of Foreign Studies, Yangtze University Jingzhou 434023 Hubei China

## Abstract

MoO_3_ is one of the most promising electrodes for high energy density supercapacitors due to its layered structure, which facilitates the insertion/removal of small ions. However, the commercial recognition of MoO_3_-based electrodes has been hampered by their low electronic conductivity, poor structural stability and narrow working potential window. A MoO_3_/C composite (MCs) has been synthesized by a polymerization method followed by calcination of the obtained hydrogel. The obtained MCs electrode exhibits remarkable electrochemical performance in both aqueous (432.5 F g^−1^ at a current density of 0.5 A g^−1^, 100% capacity retention after 10 000 cycles) and all-solid (220.5 F g^−1^ at 0.5 A g^−1^) systems with porous C as the positive electrode, demonstrating its potential in commercial utilization.

## Abstract 2

The obtained MCs electrode exhibits remarkable electrochemical performance in both aqueous and all-solid systems (220.5 F g^−1^ at 0.5 A g^−1^ with porous C as the positive electrode), certifying its excellent potential in supercapacitors.

## Graphical abstract

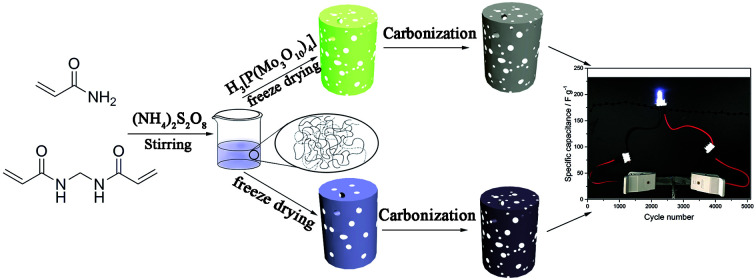

## 1. Introduction

Supercapacitors, a widely used class of energy storage devices, can perfectly bridge the performance gap between lithium ion batteries and conventional capacitors due to their advanced power density, cyclicity and excellent energy density.^1,2^ The development of supercapacitors has been spurred by the rising demand for more powerful energy devices, especially for emerging energy storage devices such as public transportation, hybrid electric vehicles, backup energy systems and consumer electronic devices. The energy density can be enhanced *via* increasing the specific capacitance (*C*) and the operation voltage (*V*) based on the equation: *E* = 1/2*CV*^2^.^3,4^ Therefore, considerable attention has been attracted by pseudo-capacitors due to their higher energy density than electrochemical double-layer capacitors resulting from their extraordinary storage mechanisms of charges, which undergo fast reversible faradaic reactions on the surface of the electrode materials.^5^

MoO_3_ has been extensively explored as a supercapacitor electrode for the improvement of energy density due to its unique structure, which is composed of stacking bilayer sheets of MoO_6_ octahedra with van der Waals interactions to facilitate the insertion/extraction of ions.^6,7^ However, MoO_3_-based supercapacitors suffer from inadequate cycle life and limited practical capacitance due to their sluggish faradaic redox kinetics, low conductivity (10^−5^ S cm^−1^) and lack of structural integrity during cycling, especially at high rates in electrochemical devices.^8,9^ Combining MoO_3_ with a conductive material appears to be an effective way to address these capacitance attenuation problems; this can significantly improve the conductivity of MoO_3_-based electrodes and provide a firm framework for MoO_3_, facilitating maintenance of the structural integrity of the electrodes during cycling. For example, Shaheen and his co-workers reported that the specific capacitance of an rGO/MoO_3_@C composite electrode can reach 562 F g^−1^ at a current density of 1 A g^−1^.^10^ Rout *et al.* synthesized a MoO_3_/reduced graphene oxide composite material which can deliver a specific capacitance of 724 F g^−1^ at 1 A g^−1^ and which exhibits a superior capacitance retention of 50% even after 800 cycles.^11^ Shakir *et al.* synthesized hydrogenated molybdenum trioxide (H_*x*_MoO_3_) nanowires that yielded a specific capacitance of 168 F g^−1^ at 0.5 A g^−1^, with excellent capacitance retention of 97% after 3000 cycles.^12^ Wen *et al.* synthesized polypyrrole@MoO_3_/reductive graphite oxide nanocomposites which demonstrate favorable cycling behavior at a current density of 0.5 A g^−1^; the capacity loss was only 12% after 600 cycles.^13^ However, the energy density of MoO_3_-based electrodes is hindered by their narrow operation potential window, which is only about −1 to 0 V *vs.* Ag/AgCl in Na_2_SO_4_ aqueous solution due to decomposition of the aqueous electrolyte. Although using organic electrolyte is a feasible approach to enhance the potential window, poor safety and environmental toxicity undermine their application potential in supercapacitors.^14–16^ Therefore, neutral aqueous electrolytes (*e.g.*, Na_2_SO_4_ and Li_2_SO_4_) are still promising electrolyte systems for the next generation of supercapacitors due to their advantages of no corrosion, environmental benignity and mass scale application.

Assembling asymmetric supercapacitors with positive electrodes provides a new cell configuration to increase the working potential window in aqueous electrolyte. Herein, we assembled an asymmetric supercapacitor using the MCs and porous carbon (PC) as a negative and positive electrode, respectively. For the negative electrode, a high surface area for faradaic reactions can be formed due to the deposition of MoO_3_ nanoparticles on the surface of the C substrate. The strong oxygen bonding between the MoO_3_ nanoparticles and carbon facilitates interfacial charge transfer and prevents the collapse of the nanostructures. For the positive electrode, the porous structure decreases the diffusion path for both ions and electrons, enhancing the rate performance of the electrode. Furthermore, considering environmental friendliness and safety, a Na_2_SO_4_/PVP gel was used as the electrolyte. Based on the working potential window of the MCs and the PC, the voltage range of the hybrid ASCs should be much wider than that of symmetric cells based on the MCs or PC. The specific capacitance of a single electrode in the ASCs can reach 220.5 F g^−1^ at 0.5 A g^−1^, certifying its outstanding electrochemical performance. Building on these data, it is reasonable to speculate that these ASCs, which can be fabricated *via* a simple procedure, hold great promise as alternative devices in energy storage applications.

## 2. Experimental

### 2.1. Chemicals and regents

Acrylamide, *N*,*N*′-methylenebisacrylamide, ammonium persulfate, phosphomolybdic acid hydrate, and ammonium hydroxide were obtained from Sinopharm Chemical Reagent Co., Ltd. No purification was performed on these chemicals before use. The resistance of the deionized (DI) water used in the reactions is 18.2 MΩ cm^−1^.

### 2.2. Preparation of MCs

For synthesis of the MCs, 5 g of acrylamide, 3.5 g of phosphomolybdic acid hydrate, 0.2 mL of ammonium hydroxide and 0.01 g of *N*,*N*′-methylenebisacrylamide were mixed in 15 mL DI water under vigorous stirring. After a homogeneous solution was formed, 5 mL of ammonium persulfate (4 mg mL^−1^) was added slowly, followed by curing at 75 °C for 5 min. The obtained hydrogel was calcinated at 650 °C for 2 h under argon after drying in a lyophilizer.

PC was synthesized through a similar synthetic procedure without addition of phosphomolybdic acid hydrate or ammonium hydroxide.

### 2.3. Characterization

The morphological details of the as-prepared samples were examined using a scanning electron microscope (SEM, Merlin, GER) at an acceleration voltage of 10.0 kV and a transmission electron microscope (TEM, a JEM-2100HR, JPN) at an acceleration voltage of 200 kV. X-ray photoelectron spectra (XPS) of the samples were obtained using an Al Kα X-ray source (1486 eV). Raman spectra were measured using a confocal LabRAM HR800 spectrometer with an excitation wavelength of 514 nm provided by an argon ion laser.

### 2.4. Electrochemical measurements

To prepare the working electrodes, the MCs (80%) or PC (80%), acetylene (10%) and polytetrafluoroethylene (10%) were mixed to form a slurry, which was dip-coated onto a carbon paper with an area of ∼1 cm^2^. After that, the obtained carbon paper was dried at 80 °C under vacuum (the mass loadings of MCs and PC were ∼1.0 mg and ∼4.8 mg, respectively). In the three-electrode configuration, the electrochemical performance of the MCs or PC was tested in 1.0 M Na_2_SO_4_ aqueous electrolyte with Ag/AgCl as a reference electrode and Pt mesh as a counter electrode. The ASC was assembled using the MCs as a positive electrode, the PC as a negative electrode, PVP/Na_2_SO_4_ gel as the electrolyte, which was prepared by mixing 4 g PVP (K30) and 3 g Na_2_SO_4_ in 40 mL DI water at 90 °C with 4 h of vigorous stirring, and fibrous paper as a separator. All electrochemical tests were carried out using a CHI 660E workstation (Shanghai Chenghua).

## 3. Results and discussion

**Fig. 1:**
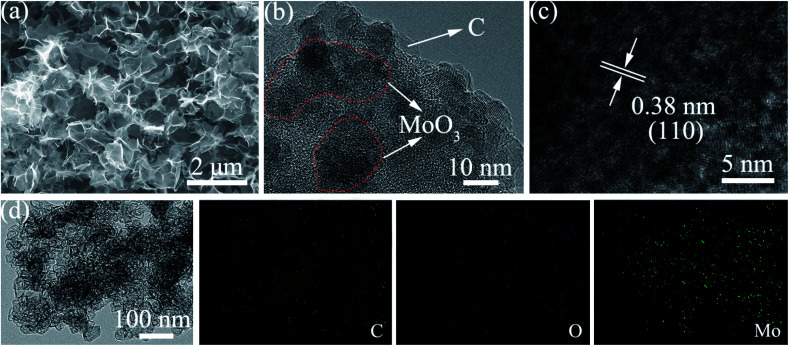
(a) SEM, (b) TEM, (c) HRTEM and (d) element mapping images of the MCs.

**Scheme 1:**
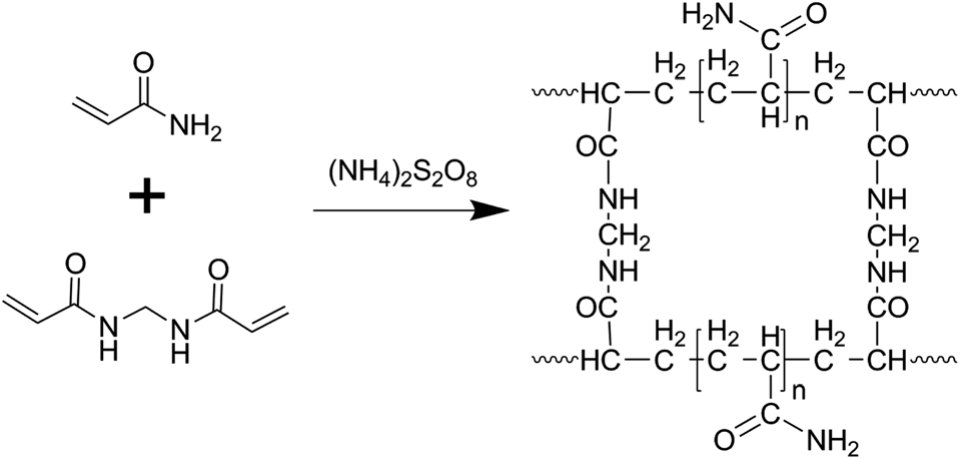
Possible polymerization reaction of acrylamide and *N*,*N*′-methylenebisacrylamide.

The morphology of the MCs is shown in Fig. 1a and b; a 3D structure with a smooth surface can be clearly observed, illustrating that the MoO_3_ nanoparticles were uniformly distributed on the surface of C. The formation of the 3-dimensional porous structure may be due to the polymerization of acrylamide and *N*,*N*′-methylenebisacrylamide (as shown in the polymerization reaction in Scheme 1). The addition of ammonium hydroxide can improve the solubility of phosphomolybdic acid hydrate in aqueous solution contained in the polymer matrix, which facilitates the even distribution of MoO_3_ in the C substrate after calcination. The existence of MoO_3_ can also be certified by HRTEM (Fig. 1c), in which well-defined lattice fringes with an interplanar spacing of 0.38 nm can be observed, matching the (110) planes of MoO_3_.^17,18^ Furthermore, element mapping analysis of Fig. 1d shows an even spatial distribution of C, O and Mo elements, which is consistent with the aforementioned measurements.

**Fig. 2:**
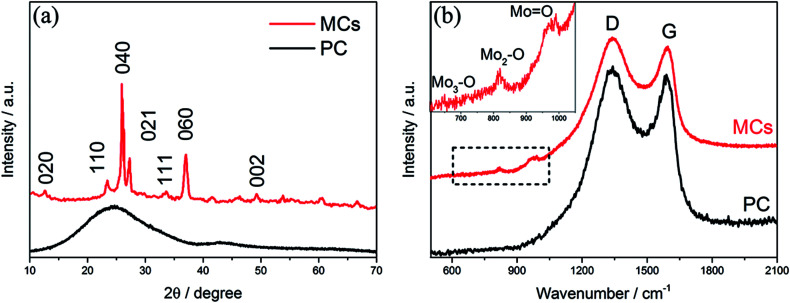
(a) XRD patterns and (b) Raman spectra of the MCs and PC.

The XRD pattern of the MCs is shown in Fig. 2a; all of the diffraction peaks can be readily indexed to α-MoO_3_ (JCPDS file no. 05-0508) with no impurities detected, suggesting high purity of the MCs.^19,20^ For the PC, only a hump diffraction peak can be observed at around 24.5°, which is ascribed to the graphite (002) plane; this results from the carbonization of the hydrogel.^21,22^ Note that the presence of MoO_3_ can also be identified by the Raman spectra in Fig. 2b; the Raman bands at around 995 cm^−1^, 818 cm^−1^ and 665 cm^−1^ are assigned to the Mo=O and Mo_3_–O stretching modes, respectively.^23,24^ The appearance of two prominent peaks in the Raman spectrum correspond to the characteristic G (associated with the E_2g_ phonons of C sp^2^ atoms) and D (arising from the mode of the *k* point phonons with A_1g_ symmetry) bands of the graphitic structure.^25,26^ The thermogravimetric analysis (TGA) curve shows that the hydrogel undergoes significant decomposition when heated to 800 °C under nitrogen flow, exhibiting a three-step degradation process, as indicated in Fig. S1.† The mass loss (∼2 wt%) below 150 °C can be attributed to removal of adsorbed water, and the mass loss (∼52 wt%) at 150 °C to 400 °C and 400 °C to 800 °C can be assigned to pyrolysis of oxygen functional groups and decomposition of phosphomolybdic acid hydrate.

**Fig. 3:**
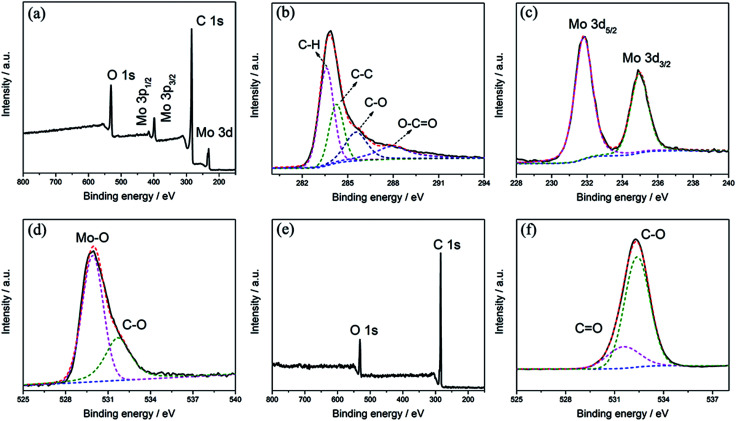
XPS survey spectrum of (a) MCs and deconvolution of the high-resolution scans of (b) C 1s, (c) Mo and (d) O. XPS survey spectrum of (e) PC and deconvolution of the high-resolution scan of (f) C 1s.

High-resolution XPS measurements were applied to characterize the chemical valence states of Mo, O and C. As shown in Fig. 3a, Mo, C and O are the only elements that can be recognized in the survey spectrum of the MCs. The deconvolution of C 1s in Fig. 3b implies the presence of four different components with binding energies of 283.8, 284.6, 285.8 and 287.9 eV, corresponding to C–H, C–C, C–O and O–C=O, respectively.^27,28^ This implies the existence of oxygenated functional groups in the C nanoparticles, which facilitates the deposition of MoO_3_ nanoparticles on the surface of the C substrate. In the spectrum of the Mo 3d region in Fig. 3c, a doublet can be identified at binding energies of 232.5 eV and 235.6 eV; these are attributed to Mo(vi) 3d_5/2_ and 3d_3/2,_ demonstrating the formation of MoO_3_.^29,30^Fig. 3d shows the spectral deconvolution of O 1s, which consists of two different components; the peak at 532.6 eV can be assigned to C–O of the C substrate, while the peak at 530.2 eV can be attributed to Mo–O in MoO_3_. The survey spectrum of the PC is shown in Fig. 3e; only C and O can be identified. As shown in Fig. 3f, two peaks at banding energies of around 531.5 and 532.4 eV can be confirmed in the spectral deconvolution of O 1s, corresponding to C=O and C–O of the C substrate, respectively.^24,31,32^ These comparisons imply the formation of C–O–Mo in the MCs.

**Fig. 4:**
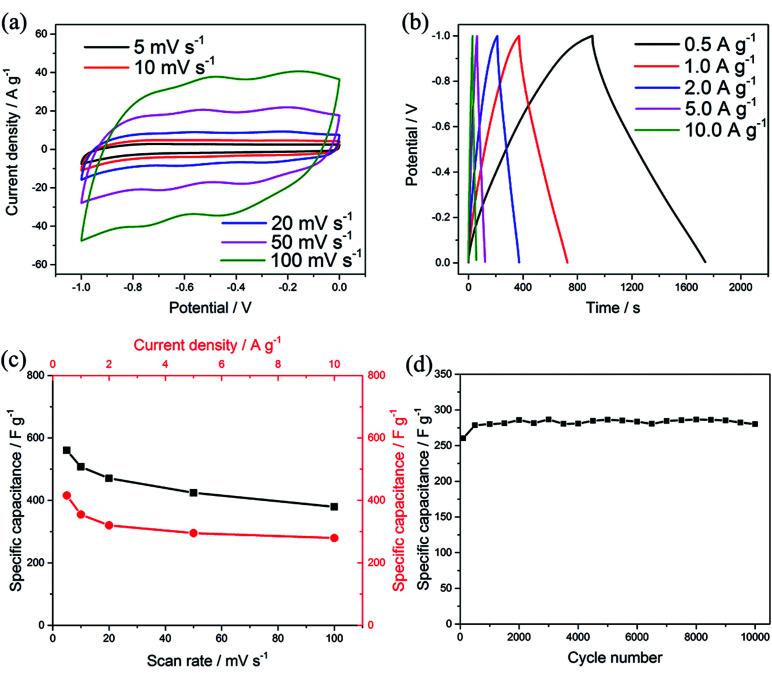
(a) CV of the MCs collected at different scan rates. (b) GCD curves of the MCs at different current densities. (c) Specific capacitances of the MCs at different scan rates and current densities. (d) Cycling life test of the MCs at a current density of 10 A g^−1^.

To evaluate the electrochemical performance of the MCs as an electrode for supercapacitors, cyclic voltammetry (CV) and galvanostatic charge–discharge (GCD) tests were performed in aqueous 1 M Na_2_SO_4_ electrolyte using a three-electrode system. Typical CV curves can be observed at a scan rate of 5–100 mV s^−1^ within a potential window of −1 to 0 V, as shown in Fig. 4a; two pairs of redox peaks can be observed at −0.21/−0.34 and −0.54/−0.68, attributed to the reversible and fast insertion/extraction of Na^+^ into/out of the MoO_3_ phase, suggesting pseudocapacitance behavior.^6,8,33^ The current response was enhanced with increasing scan rate and showed no obvious shift at the positions of the redox peaks, which is consistent with fast electron-transfer kinetics. Meanwhile, all the GCD curves presented relatively symmetric shapes with varied current densities from 0.5 A g^−1^ to 10 A g^−1^ (Fig. 4b). The specific capacitance *C* (F g^−1^) for a single electrode was calculated from the CV and GCD results by eqn (1) and (2), respectively:^34,35^1
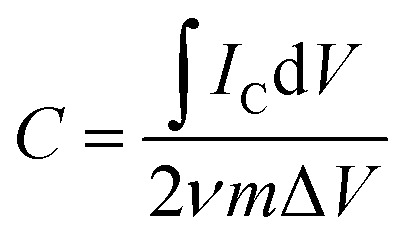
2
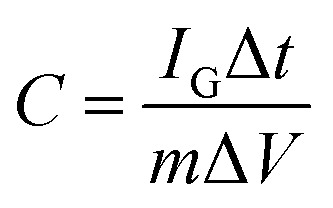
where *I*_C_ (A) is the response current, *ν* (V s^−1^) is the scan rate, *m* is the mass of the MCs-based electrode, Δ*V* (V) is the voltage window, *I*_G_ (A) is the applied discharging current, and Δ*t* (s) is the discharging time. To identify the optimal contents of MoO_3_ for high electrochemical performance, 1.5 g and 5.5 g of phosphomolybdic acid hydrate were also added; these samples were named MCs_1_ and MCs_2_. The CV curves of MCs_1_ and MCs_2_ exhibited smaller integral areas compared to that of the MCs at the same scan rate of 50 mV s^−1^ (shown in Fig. S2†); this demonstrates the optimal content of MoO_3_. Notably, the specific capacitance of the MCs shows a slight decrease from 5 mV s^−1^ (560.4 F g^−1^) to 100 mV s^−1^ (379.6 F g^−1^), suggesting superior capacity retention (Fig. 4c). Moreover, the MCs can still deliver a specific capacitance of 281.8 F g^−1^ when the current density is as high as 10 A g^−1^. The high specific capacitance is ascribed to the high specific surface area of 487.5 m^2^ g^−1^ (shown in Fig. S3†), which facilitates accessibility of the electrode to the electrolyte; meanwhile, the interconnected porous structure provides rapid and shorter diffusion paths for Na^+^. This speculation is further confirmed by the electrochemical impedance spectroscopy measurements (Fig. S4†), where the spike of the MCs shows a higher slope than that of MoO_3_ in the low-frequency region, indicating the advanced charge transfer kinetics of the MCs. The durability of the MCs-based electrode was evaluated by GCD cycling tests at a current density of 10 A g^−1^. Normally, the resistance of a layered material will increase when ions are inserted into multilayers; this may cause collapse of the structure, which may further lead to rapid attenuation of capacity. The specific capacitance presented almost no change at around 280 F g^−1^ even after 10 000 charge–discharge cycles (as shown in Fig. 4d); this indicates fast ion diffusion and excellent electronic transport, which originate from the unique 3D structure of the MCs. The C substrate in the MCs serves dual functions as both a framework to maintain the structural integrity of MoO_3_ and as conductive channels to improve the charge transfer kinetics during cycling.

**Fig. 5:**
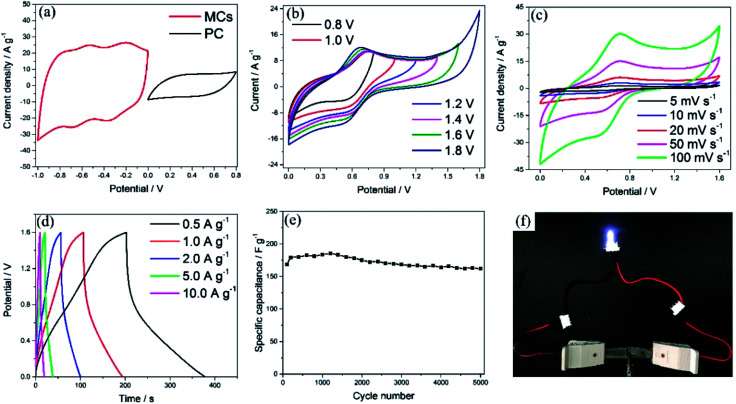
(a) CV curves of the MCs and PC at a scan rate of 50 mV s^−1^ in a three-electrode system. (b) CV curves of the ASC in different potential windows. (c) CV of the ASC collected at different scan rates. (d) GCD curves of the ASC at different current densities. (e) Cycling life test of the ASC at a current density of 2 A g^−1^. (f) Photograph of a LED lit by two devices in series.

To evaluate the electrochemical performance of the MCs in a practical cell, an ASC was assembled using PC and the MCs as the positive and negative electrode, respectively. In order to explore the stable potential windows and balance the capacity of the MCs-based electrode and PC-based electrode, CV measurements of each material were performed in 1 M Na_2_SO_4_ aqueous solution at a scan rate of 50 mV s^−1^, as shown in Fig. 5a. The PC-based electrode was measured within a potential window of 0 to 0.8 V (Ag/AgCl); it exhibited typical double-layer characteristics, which afford superior cycling performance, as shown in Fig. S5.† Meanwhile, the MCs-based electrode was measured within a potential window of −1 to 0 V (Ag/AgCl). Because the total cell voltage is the sum of the potential ranges of the positive and negative electrodes, the asymmetric cell can be operated within a potential window of 0 to 1.8 V. The specific capacitances calculated from the CV curves were 424.2 F g^−1^ for the MCs-based electrode and 110.3 F g^−1^ for the PC-based electrode. In order to balance the charges of the positive electrode and negative electrode, the optimized mass ratio of the negative electrode/positive electrode should be 0.21 in the cell, based on the equation: *Q* = *C* × *V* × *m*.^36^

CV curves of the ASC in different potential windows (from 0 to 0.8 and 0 to 1.8 V) were measured at a scan rate of 50 mV s^−1^, as shown in Fig. 5b. The distorted rectangular shapes of the CVs in different ranges of potential indicate that the capacitance of the asymmetric cell is derived from a combination of pseudocapacitance and electrochemical double layer capacitance. Moreover, no obvious irreversible current can be observed in the CV curves, even when the potential window was increased to 1.6 V; this indicates that stable performance can be achieved in this wide potential window during cycling. To assess the electrochemical performance of the ASC, CV and GCD measurements were employed, as shown in Fig. 5c and d. The current response increased with increasing scan rate in the CV curves and showed no obvious shape changes, which demonstrates the superior electron-transfer kinetics of the ASCs. The specific capacitance of one electrode in the ASCs has been evaluated from the GCD curves according to the following equation: *C* = 4*I*Δ*t*/*m*Δ*V*,^36,37^ where *I* is the applied discharge current, Δ*t* is the discharging time, *m* is the total mass of the positive and negative electrodes and Δ*V* is the potential window. A specific capacitance of 220.5 F g^−1^ was obtained at a current density of 0.5 A g^−1^, attributed to the superior electrochemical performance of both the positive and negative electrodes (which is also demonstrated by the Ragone plot in Fig. S6†). The cycling stability was evaluated between 0 and 1.6 V at a current density of 2 A g^−1^, as shown in Fig. 5e, in which the capacitance of the ASC exhibited only a slight decrease even after 5000 cycles. As shown in Fig. 5f, the ASCs were connected to a white LED to demonstrate the possibility of practical usage, and the LED could be easily lit.

## 4. Conclusions

In summary, a porous MCs-based electrode for supercapacitors has been fabricated by a simple and cost-efficient method. The MCs-based electrode exhibits remarkable electrochemical performance (432.5 F g^−1^ at a current density of 0.5 A g^−1^ and about 100% capacity retention after 10 000 cycles), which is ascribed to the 3D interconnected structure and the strong interaction between MoO_3_ and the C substrate, which facilitates good accessibility of the electrode to the electrolyte and provides rapid and shorter diffusion paths for ions. Furthermore, an ASC has been assembled using the MCs-based electrode as the anode and the PC-based electrode as the cathode; it shows a high specific capacitance of 220.5 F g^−1^ at 0.5 A g^−1^ and good capacity retention, certifying the excellent potential of the electrode for all-solid-state supercapacitors.

## Conflicts of interest

There are no conflicts to declare.

## Supplementary Material

RA-009-C8RA10232E-s001

## References

[cit1] Han Y., Lai Z., Wang Z., Yu M., Tong Y., Lu X. (2018). Chem.–Eur J..

[cit2] Zou J., Zhang M., Huang J., Bian J., Jie Y., Willander M., Cao X., Wang N., Wang Z. L. (2018). Adv. Energy Mater..

[cit3] Hou Y. N., Zhao Z., Yu Z., Zhang S., Li S., Yang J., Zhang H., Liu C., Wang Z., Qiu J. (2018). Chem.–Eur J..

[cit4] Strauss V., Marsh K., Kowal M. D., El-Kady M., Kaner R. B. (2018). Adv. Mater..

[cit5] Wang W., Zhang N., Shi Z., Ye Z., Gao Q., Zhi M., Hong Z. (2018). Chem. Eng. J..

[cit6] Chen J., Han S., Zhao H., Bai J., Wang L., Sun G., Zhang Z., Pan X., Zhou J., Xie E. (2017). Chem. Eng. J..

[cit7] Kim H. S., Cook J. B., Lin H., Ko J. S., Tolbert S. H., Ozolins V., Dunn B. (2017). Nat. Mater..

[cit8] Ruan D., Lin R., Jiang K., Yu X., Zhu Y., Fu Y., Wang Z., Yan H., Mai W. (2017). ACS Appl. Mater. Interfaces.

[cit9] Zhang S.-W., Yin B.-S., Liu C., Wang Z.-B., Gu D.-M. (2017). Chem. Eng. J..

[cit10] Shaheen W., Warsi M. F., Shahid M., Khan M. A., Asghar M., Ali Z., Sarfraz M., Anwar H., Nadeem M., Shakir I. (2016). Electrochim. Acta.

[cit11] Pathak A., Gangan A. S., Ratha S., Chakraborty B., Rout C. S. (2017). J. Phys. Chem. C.

[cit12] Shakir I., Shahid M., Rana U. A., Warsi M. F. (2014). RSC Adv..

[cit13] Yu F., Liu Y., Zhu Y., Dai F., Zhang L., Wen Z. (2016). Mater. Lett..

[cit14] Noh J., Yoon C.-M., Kim Y. K., Jang J. (2017). Carbon.

[cit15] Du P., Wei W., Liu D., Kang H., Liu C., Liu P. (2018). J. Mater. Sci..

[cit16] Qu C., Zhao B., Jiao Y., Chen D., Dai S., Deglee B. M., Chen Y., Walton K. S., Zou R., Liu M. (2017). ACS Energy Lett..

[cit17] Xiao X., Ding T., Yuan L., Shen Y., Zhong Q., Zhang X., Cao Y., Hu B., Zhai T., Gong L. (2012). Adv. Energy Mater..

[cit18] Zhou K., Zhou W., Liu X., Sang Y., Ji S., Li W., Lu J., Li L., Niu W., Liu H., Chen S. (2015). Nano Energy.

[cit19] Cao X., Zheng B., Shi W., Yang J., Fan Z., Luo Z., Rui X., Chen B., Yan Q., Zhang H. (2015). Adv. Mater..

[cit20] Zhang X., Zeng X., Yang M., Qi Y. (2014). ACS Appl. Mater. Interfaces.

[cit21] Shafi P. M., Dhanabal R., Chithambararaj A., Velmathi S., Bose A. C. (2017). ACS Sustainable Chem. Eng..

[cit22] Sari F. N. I., Ting J.-M. (2018). ChemSusChem.

[cit23] Kumar R., Goel N., Mishra M., Gupta G., Fanetti M., Valant M., Kumar M. (2018). Adv. Mater. Interfaces.

[cit24] Zhang B. Y., Zavabeti A., Chrimes A. F., Haque F., O'Dell L. A., Khan H., Syed N., Datta R., Wang Y., Chesman A. S. (2018). Adv. Funct. Mater..

[cit25] Jiang Y., Yan X., Xiao W., Tian M., Gao L., Qu D., Tang H. (2017). J. Alloys Compd..

[cit26] Chen C., Hayazawa N., Kawata S. (2014). Nat. Commun..

[cit27] Jiang Y., Jiang Z.-J., Yang L., Cheng S., Liu M. (2015). J. Mater. Chem. A.

[cit28] Briscoe J., Marinovic A., Sevilla M., Dunn S., Titirici M. (2015). Angew. Chem., Int. Ed..

[cit29] Huang L. B., Zhao L., Zhang Y., Chen Y. Y., Zhang Q. H., Luo H., Zhang X., Tang T., Gu L., Hu J. S. (2018). Adv. Energy Mater..

[cit30] Muneer M., Alam U., Bahnemann D. B. W. D., Koch J., Tegenkamp C. (2018). Phys. Chem. Chem. Phys..

[cit31] Swiatowska-Mrowiecka J., de Diesbach S., Maurice V., Zanna S., Klein L., Briand E., Vickridge I., Marcus P. (2008). J. Phys. Chem. C.

[cit32] Reddy B. M., Chowdhury B., Smirniotis P. G. (2001). Appl. Catal., A.

[cit33] Pan W., Tian R., Jin H., Guo Y., Zhang L., Wu X., Zhang L., Han Z., Liu G., Li J. (2010). Chem. Mater..

[cit34] Pham D. V., Patil R. A., Yang C.-C., Yeh W.-C., Liou Y., Ma Y.-R. (2018). Nano Energy.

[cit35] Zhang M., Chen K., Wang C., Jian M., Yin Z., Liu Z., Hong G., Liu Z., Zhang Y. (2018). Small.

[cit36] Chang J., Jin M., Yao F., Kim T. H., Le V. T., Yue H., Gunes F., Li B., Ghosh A., Xie S., Lee Y. H. (2013). Adv. Funct. Mater..

[cit37] Song P., Shen X., He W., Kong L., He X., Ji Z., Yuan A., Zhu G., Li N. (2018). J. Mater. Sci.: Mater. Electron..

