# Supplementary material for: A general method to fabricate MoO3/C composites and porous C for asymmetric solid-state supercapacitors
Source: RSC Adv. 2019 Apr 30;9(23):13207–13. doi: 10.1039/c8ra10232e (PMC9063776; doi:10.1039/c8ra10232e)
Supplement: RA-009-C8RA10232E-s001 [file RA-009-C8RA10232E-s001.pdf]

Electronic Supplementary Material (ESI) for RSC Advances.
This journal is © The Royal Society of Chemistry 2019

Supplementary material

A general method to fabricate 3D net-like MoO_3/C composite and porous C for Asymmetric Solid-State Supercapacitors

Yu Jiang [a], XueminYan[a,*], Yapeng Cheng [a], Yan Zhang [a], Wei Xiao [a], Lu Gan [c], Haolin Tang [b,*]

[a] *College of Chemistry and Environmental Engineering, Yangtze University, Jingzhou 434023, Hubei, China.*

[b] *State Key Laboratory of Advanced Technology for Materials Synthesis and Processing, Wuhan University of Technology, Wuhan 430070, PR China*

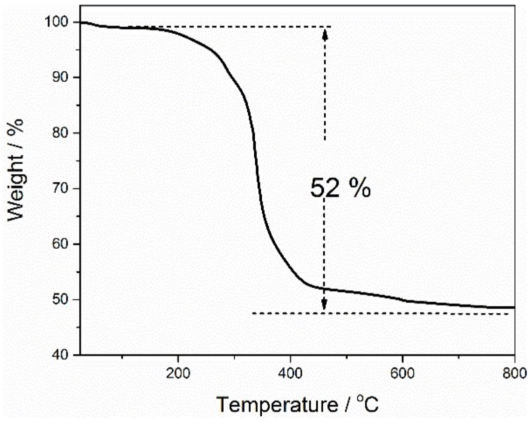

Figure S1. TGA curve of the hydrogel at a heating rate of 10 C min^{-1} under the nitrogen atmosphere.

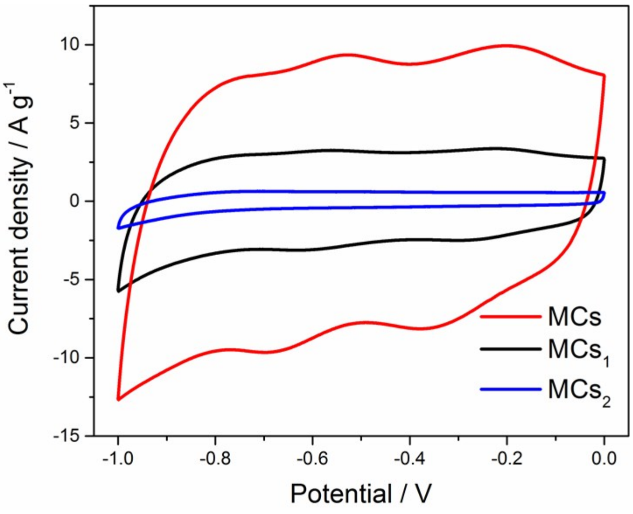

Figure S2. CV curves of the MCs, MCs_1, MCs_2 at the scan rate of 50 mV s^{-1}.

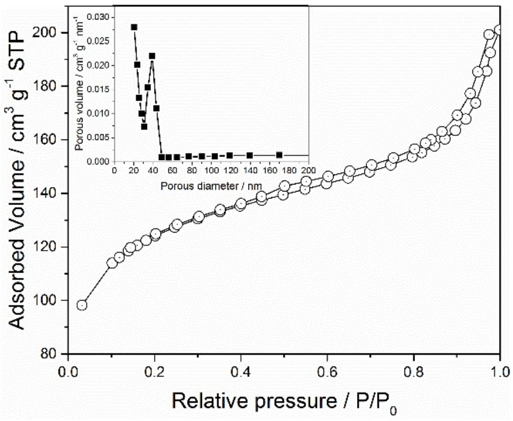

Figure S3. N_2 adsorption–desorption isotherms of the MCs. The inset picture corresponds to the BJH porous distribution of the MCs.

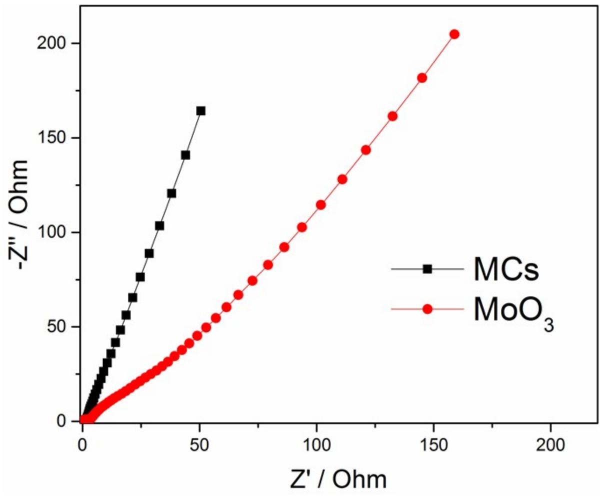

Figure S4. Nyquist plots of MCs and MoO_3. MoO_3 was fabricated by of decomposition of phosphomolybdic acidhydrate without presence of polymer matrix.

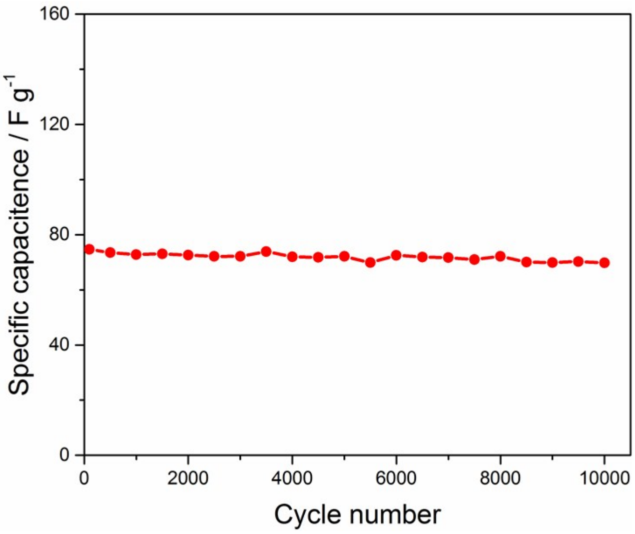

Figure S5. Cycling life test of the PC at a current density of 10 A g^{-1}.

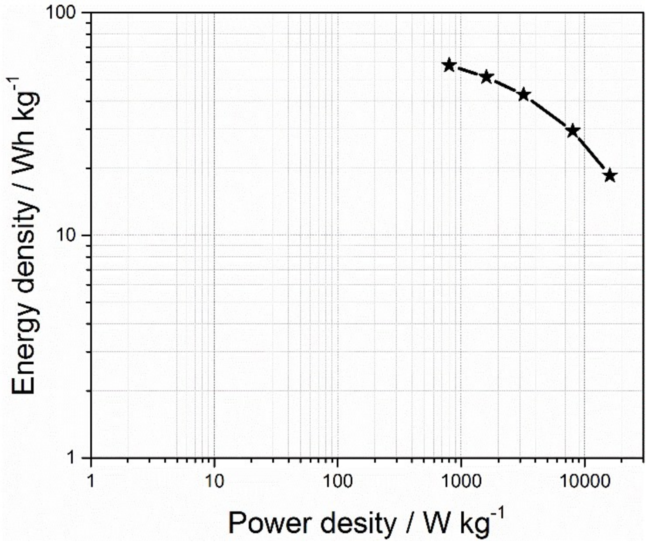

Figure S6. Ragone plots of the obtained MCs.
